# Evaluation of APP695 Transgenic Mice Bone Marrow Mesenchymal Stem Cells Neural Differentiation for Transplantation

**DOI:** 10.1155/2015/182418

**Published:** 2015-09-27

**Authors:** Qian Li, Yanjie Jia, John Zhang, Jun Yang

**Affiliations:** ^1^Department of Neurology, The Fifth People's Hospital of Chongqing, Chongqing 400062, China; ^2^Department of Neurology, The First Affiliated Hospital, Zhengzhou University, Zhengzhou 450052, China; ^3^Department of Neurology, The First Affiliated Hospital, Chongqing Medical University, Chongqing 400016, China

## Abstract

*Objective*. Even though there is a therapeutic potential to treat Alzheimer's disease (AD) with neural cell replenishment and replacement, immunological rejections of stem cell transplantation remain a challenging risk. Autologous stem cells from AD patients however may prove to be a promising candidate. Therefore, we studied the neuronal differentiation efficiency of bone marrow mesenchymal stem cells (MSCs) from APP695 transgenic mice, which share features of human AD. *Method*. Cultured MSCs from APP695 transgenic mice are used; neuronal differentiation was assessed by immunocytochemistry and Western blot. Correlation with Notch signaling was examined. Autophage flux was assessed by western blot analysis. *Results*. MSCs from APP695 mice have higher neuronal differentiation efficiency than MSCs from wild type mice (WT MSCs). The expression of Notch-1 signaling decreased during the differentiation process. However, autophagy flux, which is essential for neuronal cell survival and neuronal function, was impaired in the neuronally differentiated counterparts of APP695 MSCs (APP695 MSCs–n). *Conclusion*. These results suggested autologous MSCs of APP690 mice may not be a good candidate for cell transplantation.

## 1. Introduction

Alzheimer's disease (AD) is the most common form of dementia, characterized pathologically by the presence of large numbers of neuritic plaques, neurofibrillary tangles, and a massive loss of neurons, especially cortical and hippocampal neurons [1, 2]. An emerging potential treatment option for AD is stem cell transplant. Bone marrow mesenchymal stem cells (MSCs) are multipotent nonhematopoietic cells with the capacity for differentiation into neural cells [3]. MSCs with neurogenic potentials are found in the hippocampus and the subventricular zone, which are two of the mainly affected regions of the AD brains. To overcome the potential immunological rejections to the stem cells, autologous MSCs may be a good candidate for cell transplantation for AD patients [4].

APP is a single transmembrane protein with a long N-terminal domain and a short cytoplasmic tail [5]. Recent studies have shown that APP promoted differentiation of pluripotent stem cells toward a neural fate [6]. Proteolysis of APP such as sAPPa, sAPP*β*, and even A*β* is also found to be neuroprotective. They stimulate proliferation of adult neural progenitors and even promote stem cells differentiation into neurons [7].

Notch signaling plays an important role in the development of the nervous system, including regulation of neural stem cell (NSC) proliferation, self-renewal, differentiation, and other biological activities [8]. APP and Notch are both processed by *γ* secretase [9]. It is unclear whether there is a cross-talk between APP and Notch signaling pathways in the process of MSC neuronal differentiation.

Autophagy is a nonselective degradation pathway by which long-lived proteins and organelles are sequestered in autophagosomes and degraded upon their fusion with lysosomal components [10]. Autophagic vacuoles have been found to accumulate in dystrophic neuritis and in the cell body of the AD brain, which have been shown partly accountable for the overproduction of A*β* [11]. Abnormal accumulation of autophagic vacuoles resulted from impaired autophagic vacuoles maturation to lysosomes [12]. Therefore, we need to address before testing the possible therapeutic use of autologous MSCs of AD patients and whether neuronally differentiated counterparts have deficiency in autophagy, which would jeopardize the benefits of autologous MSCs transplantation.

In this study, we investigated whether the Notch signaling is involved in the neuronal differentiation of APP695 MSCs and whether autophagy flux is impaired in the neurons differentiated from APP695 MSCs.

## 2. Materials and Methods

### 2.1. Cell Culture

APP695 transgenic mice overexpressing 695 human amyloid precursor protein (hAPP) carrying Swedish familial mutation (K670N/M671L) [13] were obtained from the Institute of Laboratory Animal Sciences, the Chinese Academy of Medical Sciences and Peking Union Medical College (CAMS and PUMC). The wild-type littermates with the same genetic background were used. All animal procedures used in this study were approved by the Institutional Animal Care Committee of Zhengzhou University, China. MSCs were isolated from femurs and tibias of mice, the cells were then plated onto culture plates in a complete medium consisting of Dulbecco's Modified Eagle's Medium (DMEM) (Invitrogen) and 10% fetal bovine serum (Invitrogen) and incubated in a 37°C, 5% CO_2_ incubator. The cells were harvested with 0.25% trypsin + 0.04% ethylenediamine tetraacetic acid (EDTA) when the confluence reached 80–90%. The cells were cultured for five passages before being used for assays.

### 2.2. Differentiation into Neurons In Vitro

The MSCs were divided into two groups: the APP group of MSCs from APP transgenic mice and the WT group of MSCs from wild-type mice. When MSCs grew to 50%~70% confluence, cells were induced by wiping off DMEM and rinsing three times with phosphate-buffered saline (PBS), and then cultured in DMEM containing 10% fetal bovine serum and 1 mM *β*-mercaptoethanol (*β*-ME) for 24 hours. The cells were then transferred to serum-free medium containing 10 mM *β*-ME for 5 d.

### 2.3. Amyloid-*β* Level Assay

The cell culture medium was collected. The A*β* 40 and A*β* 42 levels were measured by following the manufacturer's protocols of mouse amyloid *β* peptide A*β* 40 and A*β* 42 enzyme-linked immunosorbent assay Kits (R&D, Minneapolis, Minnesota, USA).

### 2.4. Immunocytochemistry

After washing with PBS, the cells were fixed for 10 minutes at −20°C in 100% methanol and then incubated in 1%BSA/10% normal goat serum/0.3 M glycine in 0.1% PBS-Tween for 1 hour to permeabilize the cells and block nonspecific protein-protein interactions and subsequently incubated with primary antibodies overnight at 4°C, including MAP-2 (Santa Cruz), NSE (Santa Cruz), and LC3B (Cell Signaling) at 4°C. After three washes in PBS, the cells were incubated with the secondary antibody (anti-Ig-G-Cy3 goat anti-rabbit, Santa Cruz) at room temperature for 2 hours. The cells were visualized using a Carl Zeiss confocal microscope.

### 2.5. Western Blot Analysis

Cells from each group were washed one time with PBS and lysed in RIPA buffer; then the samples were centrifuged at 15 000 ×g for 10 min and the supernatants were collected and stored at −80°C. An equal amount of cell lysate was separated by SDS-PAGE and then transferred to polyvinylidene fluoride (PVDF) membrane. The membrane was then blocked with TBST containing 5% nonfat milk for 2 hours at room temperature, followed by incubation with primary antibodies LC3B (Cell Signaling), MAP-2 (Santa Cruz), NSE (Santa Cruz), Notch-1 (Santa Cruz), NICD (Santa Cruz), Hes5 (Santa Cruz), and *β*-actin (Santa Cruz) overnight at 4°C and then incubated with horse radish peroxidase-conjugated anti-rabbit IgG (1 : 2000, Santa Cruz) for 2 hours at room temperature. Detection of reactive antigens was performed using an ECL kit (Santa Cruz).

Autophagy Flux Assay: When induction was finished, differentiated APP MSCs and differentiated WT MSCs were removed to neural culture media (neurobasal-A media (Invitrogen) containing 2 mM GlutaMAX-I Supplement, 2% B27, and 100 *μ*/mL penicillin/streptomycin) plus 10 *μ*mol/L rapamycin to induce autophagy. The levels of p62 at different time points (0 hours, 6 hours, and 12 hours) were analyzed by Western blot.

### 2.6. Statistical Analysis

All data are expressed as means ± SD. To determine whether a difference was significant, variance analysis was used between the groups, and the results of different groups were compared using Student's *t*-test. The differences were considered significant if *P* < 0.05.

## 3. Results

### 3.1. APP MSCs Had Higher Neuronal Differentiation Efficiency

**Figure 1:**
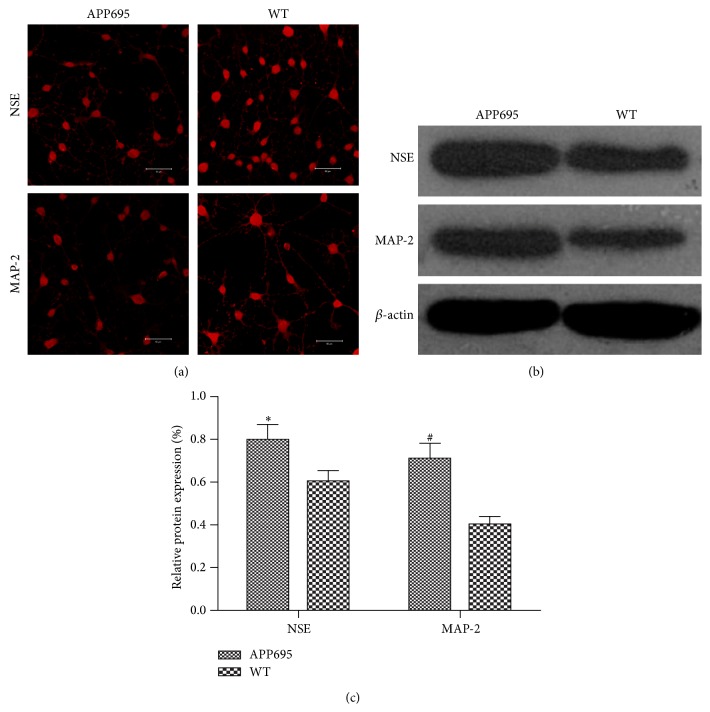
APP695 MSCs had higher neuronal differentiation efficiency. The expression of NSE and MAP-2, 6 days after induction with *β*-ME. (a) The expression of MAP-2 and NSE by immunocytochemical staining is shown (scale bar: 20 *μ*m). (b) The expression levels of NSE and MAP-2 in MSCs 6 days after induction with *β*-ME as analyzed by western blot. (c) The quantification of the expression levels of NSE and MAP-2 (mean ± SD; *n* = 6). APP695 versus WT, ^*^ *P* < 0.01 for NSE, and ^#^ *P* < 0.01 for MAP-2.

MSCs were induced by *β*-mercaptoethanol for six days, the morphologies of MSCs began to change after 24 hours induction. Some cells contracted their cytoplasm into globular or spindle-shaped bodies and emitted cellular processes, and the majority of the cells became typical neuron-like cells (Figure 1(a)). To measure the neuronal differentiation efficiency, immunocytochemistry and Western blotting were applied to analyze the expression of neuronal markers NSE and MAP-2 in each group. There were higher expressions of NSE and MAP-2 in cells from the APP695 group than in the wide type mice group (Figures 1(b)-1(c)).

### 3.2. Secretion of A*β*40 and A*β*42 from Differentiated MSCs

The results of the enzyme-linked immunosorbent assay showed that after a five-day induction, the level of A*β*40 in the induction medium of APP group was 23.2 ± 3.5 pg/mL and the level of A*β*42 in the induction medium of APP group was 3.3 ± 0.6 pg/mL. A*β*40 and A*β*42 were not detected in the induction medium of WT group.

### 3.3. Notch Signaling Was Inhibited during Differentiation of the MSCs

**Figure 2:**
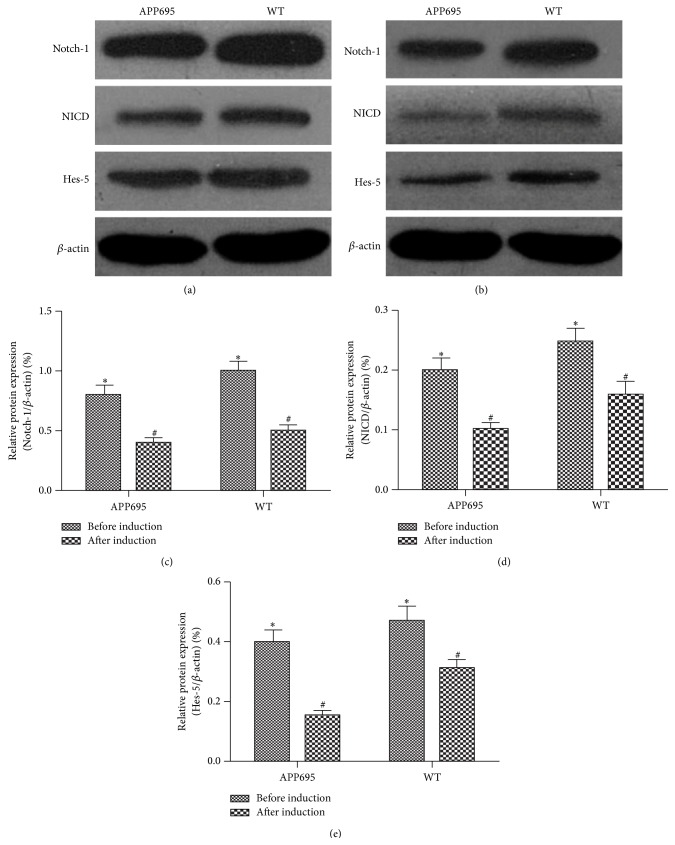
Notch signaling was inhibited during differentiation of the MSCs. (a) The expression levels of Notch-1, NICD, and Hes5 were analyzed by western blot before the induction with *β*-ME. (b) The expression levels of Notch-1, NICD, and Hes5 after the induction with *β*-ME. The expression of *β*-actin was used as a loading control. (c, d, and e) The quantification of the expression levels of Notch-1, NICD, and Hes5 (mean ± SD; *n* = 6). APP695 versus WT (before induction), ^*^ *P* < 0.05; APP695 versus WT (after induction), ^#^ *P* < 0.05.

To investigate the effect of Notch signaling during the differentiation of the MSCs, the expression of Notch-1, NICD, and Hes5 was measured by western blot before (Figure 2(a)) and after (Figure 2(b)) differentiation induction. The results from western blot showed the expression levels of Notch-1, NICD, and Hes5 significantly decreased after the induction of differentiation in both groups, particularly in APP695 group (Figure 2(c)).

### 3.4. Autophagy Flux Was Impaired in APP695 MSCs-n

**Figure 3:**
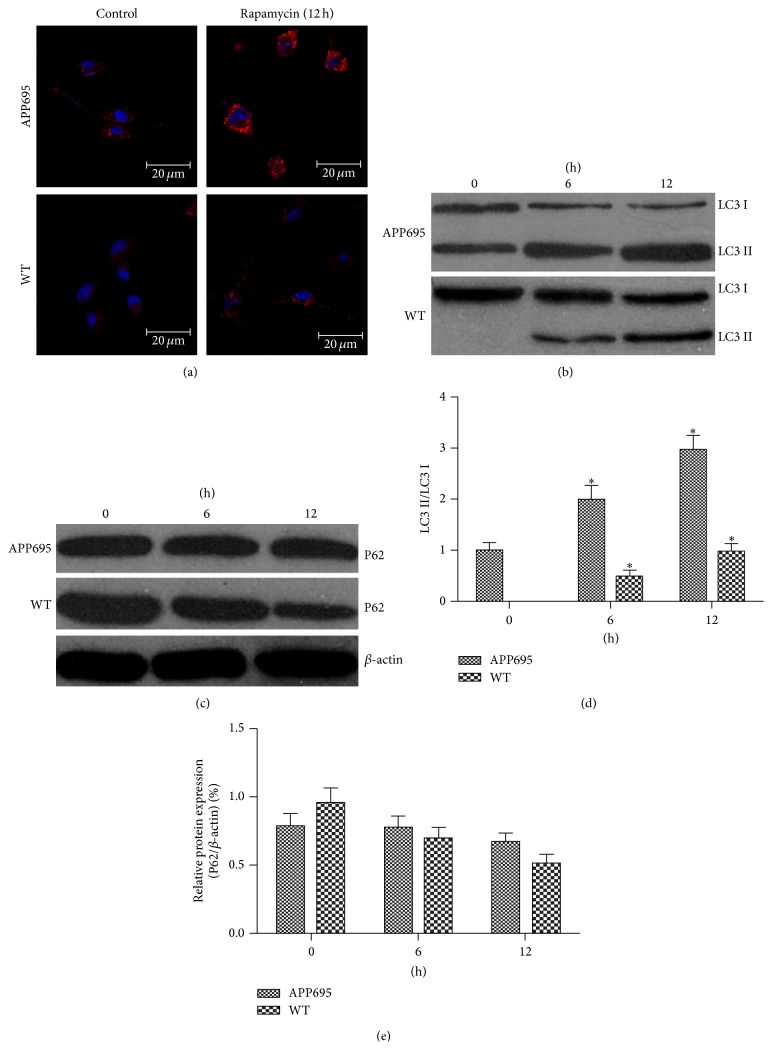
Autophagy flux was impaired in APP695 MSCs. APP695 and WT MSCs-n (neuronally differentiated counterparts of APP695 MSCs) were cultured in complete medium plus 10 *μ*mol/L rapamycin for the indicated times and then subjected to immunocytochemical staining (a) and western blot (b, d) using anti-LC3 antibody. (c, e) Cells were cultured as in (a) and p62 expression levels were analyzed by Western blot. LC3 II/LC3 I ratio increased in both APP695 and WT cells (^*^ *P* < 0.05 versus zero hr) but more pronounced in APP695 (^#^ *P* < 0.05 versus WT).

To evaluate the autophagy activity in APP695 MSCs-n (neuronally differentiated counterparts of APP695 MSCs), Western blot and immunocytochemical staining were used to measure the expression of LC3 I-II, which is closely correlated with the number of autophagic vacuoles (AVs), serving as a good indicator of AVs formation [14]. To distinguish whether the AVs accumulation was due to autophagy induction or rather a block in downstream steps, “autophagic flux” assays were used. LC3 II and fluorescent dots increased during the autophagy activated by rapamycin in both groups (Figure 3(a)). The results of western blot showed there was a higher expression of LC3 II in APP695 MSCs-n and fluorescent dots (AVs) accumulated in APP695 MSCs-n (Figure 3(b)). While no apparent changes were observed in the level of P62, a selective substrate of autophagy, in APP695 MSCs-n, a slight, but not significant decrease of P62 occurred 12 hours after differentiation induction in WT MSCs-n from wide type mice (Figure 3(c)). The ratio of LC3 II/LC3 I demonstrated a time dependent increase over differentiation induction (*P* < 0.05 versus zero hour, Figure 3(d)) in MSCs from APP695 than cells from wide type (APP695 *P* < 0.05 versus WT). However, the levels of P62 are not different between APP695 and wide type (*P* > 0.05, Figure 3(e)).

## 4. Discussion

This study showed an increased differentiation in MSCs from APP695 than MSCs from wide type mice, the expression of neuron-specific markers, MAP-2 and NSE was higher in APP695 MSCs than wide type MSCs, and indicating APP695 MSCs were more inclined to differentiate into neuronal cells. Notch-1, NICD, and Hes-5 signals were decreased after differentiation, especially in APP695 MSCs. Autophage flux seemed more pronounced in APP695 MSCs than those from wide type mice.

APP and its role in AD have been established, in that APP has a short half-life and is metabolized by two distinct antagonist pathways, resulting in cleavage by *α* secretase to generate sAPPa or *β* secretase to generate sAPP*β*. A*β* peptide and the intracellular domain (AICD) were released by additional *γ* secretase cleavage following *β* secretase cleavage [7]. The formation of A*β* and its subsequent deposition in senile plaques are regarded as the initial pathological changes resulting in AD [15]. However, recent studies suggest that A*β* may have positive effects besides deleterious actions [16]. A*β* peptide can increase the total number of neurons instead of impairing the neurogenic rate in NSC progeny [17, 18]. A*β*1–42 treatment stimulated neurogenesis of subventricular zone precursors in young adult through the p75 neurotrophin receptor [19]. In the present study, secretion of A*β* from APP695 MSCs-n was detected, whereas none was detected from WT MSCs-n. This might contribute to the higher neuronal differentiation efficiency of APP695 MSCs. APP*α* and sAPP*β* have similar properties: they are neuroprotective and they promote neurite outgrowth. sAPP*α* and sAPP*β* had also been shown to induce human embryonic stem cells to differentiate into neurons [6].

AICD, the intracellular domain of APP, binds to the cytosolic adaptor proteins Numb and Numb-like (Nbl), known inhibitors of Notch signaling, and inhibits NICD [20]. Kim et al. found AICD accelerated degradation of the Notch1 intracellular domain (Notch1-IC) and RBP-Jk. It also suppressed Notch1 transcriptional activity by the dissociation of the Notch1-IC–RBP-Jk complex, so AICD functions as a negative regulator in Notch1 signaling [21]. The Notch signaling is one of the pathways regulating cell fates, cell proliferation, and cell death in developmental stage [22]. Notch functions as a receptor and mammals have four Notch receptors (Notch1, Notch2, Notch3, and Notch4), many ligands, and downstream target genes [23]. Hes-5 is one of the downstream target genes, which is one of the key regulators of NSC proliferation and differentiation [24].

Notch signaling inhibits neuronal differentiation and guarantees the successive waves of neurogenesis from neural stem/progenitor cell pool [25]. Deletion of Rbpj in the embryonic brain, which is an intracellular signal mediator of all Notch receptors, resulted in all telencephalic neural stem/progenitor cells prematurely differentiated into neurons [26]. In our previous studies, we reported that the Notch signaling played a negative role in MSC differentiation into neural cells [27]. In this study, we found the expression of Notch-1, NICD, and Hes5 decreased in both groups after differentiation induction. The greater suppression was in the APP695 group, which indicated the Notch signaling pathway was inhibited during the differentiation process.

Neurons have highly dynamic cellular processes for their proper functions such as cell growth, synaptic formation, or synaptic plasticity by regulating protein synthesis and degradation [28]. Autophagy is an intracellular degradation process that clears long-lived proteins and organelles from the cytoplasm. Therefore, autophagy is the main quality control of proteins mechanism in neurons, which is essential for their physiology and pathology [29]. Autophagy is extensively involved in the neurodegenerative/regenerative process in AD patients. Autophagic vacuoles (AVs) were abundant in AD brains particularly, within neuritic processes, including synaptic terminals [30]. Abundant AVs resulted from impaired clearance of AVs [31]. AVs are a previously unrecognized and potentially highly active compartment for A*β* generation and this indicated that the abnormal accumulation of AVs in affected neurons of the AD brain contributes to *β*-amyloid deposition [11]. In the present study, AVs accumulated in APP695 MSCs-n were observed and there was a partial block in autophagosomal maturation and the completion of the autophagy pathway in the APP695 MSCs-n. These observations indicate a potential risk for transplantation using autologous MSCs.
